# Consumer Grade Weather Stations for Wooden Structure Fire Risk Assessment

**DOI:** 10.3390/s18103244

**Published:** 2018-09-27

**Authors:** Torgrim Log

**Affiliations:** Department of Fire Safety and HSE Eng., Glö∂ R&D, Western Norway University of Applied Sciences, 5528 Haugesund, Norway; torgrim.log@hvl.no; Tel.: +47-900-500-01

**Keywords:** relative humidity, consumer grade weather stations, calibration, winter fire risk

## Abstract

During January 2014, Norway experienced unusually cold and dry weather conditions leading to very low indoor relative humidity (RH) in inhabited (heated) wooden homes. The resulting dry wood played an important role in the two most severe accidental fires in Norway recorded since 1923. The present work describes testing of low cost consumer grade weather stations for recording temperature and relative humidity as a proxy for dry wood structural fire risk assessment. Calibration of the weather stations relative humidity (RH) sensors was done in an atmosphere stabilized by water saturated LiCl, MgCl_2_ and NaCl solutions, i.e., in the range 11% RH to 75% RH. When calibrated, the weather station results were well within ±3% RH. During the winter 2015/2016 weather stations were placed in the living room in eight wooden buildings. A period of significantly increased fire risk was identified in January 2016. The results from the outdoor sensors compared favorably with the readings from a local meteorological station, and showed some interesting details, such as higher ambient relative humidity for a home close to a large and comparably warmer sea surface. It was also revealed that a forecast predicting low humidity content gave results close to the observed outdoor weather station data, at least for the first 48 h forecast.

## 1. Introduction

Fire is a major cause of accidental injury and results in over 300,000 deaths annually [1,2,3]. The fires are usually associated with combustible spill accidents and fires in hot climates. Recently, subzero-temperature fires have, however, caught increased attention from researchers who have found these fires to be extremely severe and fast developing [4]. The Lærdalsøyri fire in Western Norway 18–19 January 2014 destroying 40 buildings and threatening the whole village including the historical Old Lærdalsøyri [5], may serve as an example. Ten days later, the Flatanger wild fire resulted in the loss of 60 structures in the Trøndelag region, a short distance south of the Arctic Circle [6].

In these areas, as well as in the majority of the country, wooden homes dominate the building style due to the abundance of wood as a construction material. One of the precursors for the severe fires was ambient subzero temperature air of low relative humidity for a few weeks before these fires. This resulted in dry outdoor combustible materials and extremely dry indoor wood in inhabited (heated) wooden structures [5,6]. A correlation between urban building fire frequency and low dew point temperature during winter time for selected areas in the USA was demonstrated as early as in 1956 by Pirsko and Fons [7]. It is also generally known that building fires are more common during winter in cold climates [8]. To monitor the temperature and the ambient relative humidity outdoor, as well as indoor, may then represent an indirect way of monitoring gradually changing winter fire risk. The wind plays a major role in the fire spread between buildings. The weather forecasts for the next days in combination with the monitored indoor RH may then in the future probably be used to design a conflagration fire danger rating system [9].

However, such an application reveals the need for low cost sensors for recording ambient and indoor temperature and RH. Recently, a fast TiO_2_ capacitive high speed sensor with 35 s response time [10] and an extremely fast response induced stress-optic polymer fiber sensors with only 2 s response time [11] have been developed. The relative humidity indoors does, however, change slowly. A one to two hour response time would be satisfactory at least for testing the concept in the present study. To assist in data collection, it would be preferable to use equipment that allows for remote access of the recorded data through the internet for analysis and risk evaluation. Possible warnings to the fire brigades in case of unusual dry conditions developing could then be issued. It was therefore considered to determine whether comparably low cost consumer grade weather stations could be used for relative humidity measurements as a proxy for increased fire risk.

The objective of the present work is to report on the experience of testing consumer grade weather stations for relative humidity measurements as a proxy for the winter fire risk in wooden constructions (Section 1). Section 2 presents the theoretical background. Section 3 describes the need for calibration and a simple way to establish calibration curves without a professional climate chamber. Section 4 presents the results regarding the main objective as well as some other interesting findings. In Section 5 the overall experience with the consumer grade weather stations for scientific measurements are discussed. Suggestions for improvement and future research are also presented.

## 2. Theory

### 2.1. The Relative Humidity Conten in Air

The relative humidity in air is dependent on the absolute water vapor content as well as the air temperature. The saturation vapor pressure of water is a near exponential function of temperature and may be described by [12]:

(1) $$P_{sat}=610.78\cdot \exp\left(\frac{17.2694\cdot T_{o}}{T_{o\;}+\;238.3}\right)\;\left(\mathrm{Pa}\right),$$

where $T_{o}$ (°C) is the ambient temperature. When the temperature and the relative humidity of the air are known, the water vapor concentration may be calculated by:

(2) $$C_{w,o}=RH_{o}\cdot \frac{P_{sat}\cdot M_{w}}{R\cdot T_{o}},\left({\mathrm{kg}\;m}^{-3}\right)\;,$$

where $RH_{o}$ is the ambient relative humidity (in the range 0 to 1.0), $M_{w}$ (0.01802 kg mol^−1^) is the molecular mass of water and R (8.314 J K^−1^ mol^−1^) is the molar gas constant.

Cooling this air so that it theoretically contains more than 100% relative humidity, the surplus water condenses at small dust particles, etc. to make fog. It is a quite normal phenomenon to observe fog in a cold night and morning dew on the grass after a cold night. It should be noted that cooling the air results in a volume reduction, which also increases the water vapor concentration. The focus of the present paper is, however, the indoor heating of the air in cold climates. Heating the cold ambient air (at constant pressure) to the higher indoor temperature, $T_{in}$ (°C), will, according to the ideal gas law, result in a dilution corresponding to the gas volume expansion, i.e.,

(3) $$C_{w,in}=C_{w,o}\cdot \left(\frac{T_{o}\;+\;273.15\;K}{T_{in}\;+\;273.15\;K}\right)\left({\mathrm{kg}\;m}^{-3}\right).$$

### 2.2. Concentration of Water in the Air

In Norway, historic weather data at 1 h frequency may be retrieved for free from a number of meteorological stations by the application provided at www.eklima.no. In the Haugesund area, the meteorological station at the Haugesund airport is well equipped and professionally maintained for recording data relevant for the present study. Based on retrieved temperature and relative humidity data, the historic values for the ambient water vapor concentration may be calculated by using Equations (1) and (2).

**Figure 1:**
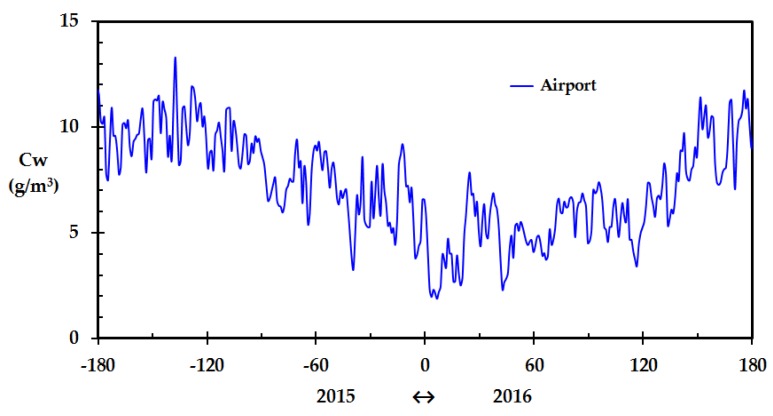
Calculated outdoor water concentration based on temperature and relative humidity recorded by the meteorological station (Haugesund airport), July 2015 through June 2016. (The x-axis represents days prior to and after New Year).

The calculated outdoor water concentration based on data from the meteorological station at the Haugesund airport for the period July 2015 through June 2016 is shown in Figure 1. The variation through the year is significant. With a few exceptions, it is seen that the low water content in parts of January, i.e., about 2 g/m^3^, was indeed very low compared to the rest of the year. If such dips are short, i.e., less than a day, a wooden home does not have time to adjust to the new dry conditions. This is partly due to the finite air exchange rate as well as slow diffusion processes of water in wood and humidity being released from the drying wood to the indoor air. However, if the dry conditions persist for some days or weeks, the wood dries out and the fire risk related to the drier wood increases [5]. In order to analyze this situation, information about the indoor relative humidity is needed. Preferably, this should be recorded in a cost efficient and convenient way, e.g., through the internet.

### 2.3. Moisture Supply Sources Indoors

Indoors, there are usually also sources of moisture supply present, e.g., humans, pets and pot plants. Hygroscopic materials, e.g., wood and other cellulose based materials such as upholstery, clothes, carpets, etc. may also release humidity when the indoor climate gets drier. If the indoor air gets more humid, these materials absorb humidity from the air. These materials may also show hysteresis when changing from the mode of adsorption to desorption and vice versa [13]. Since such hysteresis is dependent on parameters such as thickness, internal humidity diffusion processes, etc. of the variety of materials involved, it is quite complicated to model the contribution of these materials to the indoor humidity levels.

The best way to measure the water content of indoor combustible hygroscopic materials would probably be to record the mass change of selected objects. This is not easy to do with a good precision. Measuring the indoor relative humidity does, however, provide a good alternative for obtaining information about the indoor climate contribution to structural wood fire risk. Recording the humidity levels, and keeping track of especially low indoor humidity levels, gives a good indication about the structural wood fire risk development. Warnings may be considered based on weather forecasts so that focus can be shifted to monitoring indoor relative humidity development and fire risk evaluations when needed.

## 3. Materials and Methods

### 3.1. Initial Considerations and Preliminary Testing

The first idea was to record the mass of representative indoor wooden objects in order to consider their combustibility. This could be e.g., wooden plates of different thicknesses placed on separate balances, and then record the mass at a convenient frequency. The recorded data could then be transferred via the internet to researchers analyzing the data. It was, however, realized that it would be quite challenging to e.g., have sufficiently stable balances. A number of precision balances would also have been very costly. During discussions with an automation engineer [14], it was decided to try consumer grade weather stations for recording relative humidity as a proxy for the indoor wood fire risk. It was therefore decided to abandon the direct mass recordings and further pursue the weather station proposal.

A very low cost, and not web based, weather station was therefore purchased and evaluated against a precision psychrometer (Extec RH390, ID 10116891, calibration certificate 350342-10116891, Extech Instruments, Waltham, MA, USA). The psychrometer calibration data are presented in Table 1. A straight line was fit to the RH390 psychrometer RH readings as a function of the climate chamber RH to give:

*RH* = 1.0345·*RH*_reading_ − 0.5683, (4)

where the deviations are stated in the last column of Table 1.

The weather station and the precision psychrometer were placed in a 17 L transparent plastic box with a lid. Operation of the precision psychrometer without disturbing the internal atmosphere was provided for by a 2.5 mm plastic rod through a sealed hole. The internal relative humidity was adjusted to get varying relative humidity (RH) in the plastic box. To get a low RH, the box was taken outdoor and filled with low temperature air which became dry when adjusted to indoor conditions at 22 °C. To get high RH, droplets of water were allowed to evaporate in the plastic box, inspired by a previous study [15]. Comparing the results recorded by the very inexpensive weather station with the results obtained by the precision psychrometer, a simple straight line, similar to the one described by Equation (4), was obtained for the deviation. Subsequent recordings corrected by this straight line gave results within the psychrometer precision, i.e., within 2% RH. The response time and longtime stability of this inexpensive weather station were not further investigated. The results obtained were, however, very promising regarding the potential of using more advanced consumer grade weather stations. It was therefore decided to proceed with a higher quality weather station, which had a built in system for web based data transfer.

### 3.2. The Netatmo Weather Stations

**Figure 2:**
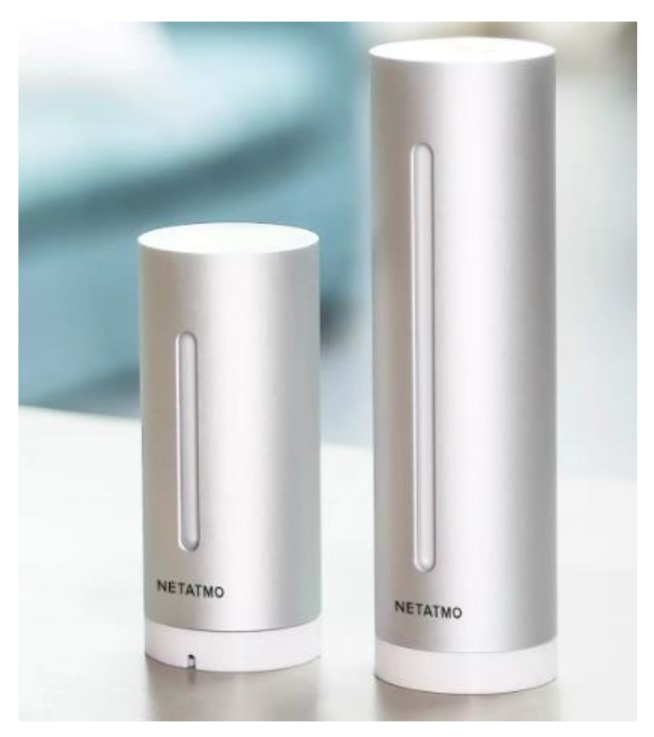
Netatmo weather station outdoor unit (**left**) and indoor unit (**right**). (Photo by Netatmo, reproduced with permission).

Eight Netatmo weather stations (Netatmo Urban Weather Station, Wi-Fi, NWS01-EC, [16]), as shown in Figure 2, were purchased from a local hardware shop. These units were set up to the local WiFi network by a PC for later data access by smartphones and PCs through a password protected WiFi connection. These weather stations record temperature and relative humidity at 5 min intervals. The indoor unit also records atmospheric pressure and CO_2_-concentration. The outdoor unit sends the recorded data to the indoor unit, which automatically forwards the recorded indoor and outdoor data to the Netatmo servers for instant or later user access and data retrieval. The complete weather station, including indoor and outdoor units, is shown in Figure 1. The indoor units are powered by a USB adapter while the outdoor units are battery powered. The outdoor sensor battery status is transmitted to the user web page. It is also possible to give public access to temperature and RH recordings. This service is currently being used to improve the temperature predictions of the Norwegian weather forecasts [17].

The producer of the Netatmo units states that the equipment can read temperatures within ±0.5 °C and relative humidity (RH) within ±3% RH. The PC interface allows for a temperature calibration by the user. In the present work it was decided to calibrate the temperature output of the units against a precision temperature instrument (ALMEMO^®^ SP10302D Pt100 Temperature Reference Instrument, Ahlborn Mess- und Regelungstechnik GmbH, Holzkirchen, Germany) for the temperature interval relevant for the indoor and outdoor sensors. Only minor adjustments were needed to achieve temperature recordings well within ±0.3 °C for the temperature region of interest in the present work.

There was, however, no web interface available for the user to calibrate the RH readings. The producer of the Netatmo units only provides a single point adjustment at about 75% RH. When testing the readings of two Netatmo indoor and outdoor units against the precision psychrometer, a clear need for calibrating the weather station indoor and outdoor readings was revealed. It was therefore decided to obtain calibration curves for all indoor and outdoor units.

### 3.3. The Equipment and Chemicals Used for RH Calibration

To make an atmosphere of known and constant temperature and relative humidity (RH), the sensors to be calibrated were placed, four at a time, in a transparent plastic box of dimensions 50 cm by 40 cm by 30 cm height. A lid was applied to the box and the USB power cables for the indoor units were let out through the back wall. The hole was sealed to prevent air exchange with the surroundings.

Water saturated inorganic salt solutions, i.e., LiCl, MgCl_2_ and NaCl, all salts pro analysis quality, were used to respectively achieve RH values of 11.3 ± 0.31% RH, 33.07 ± 0.18% RH and 75.47 ± 0.14% RH [18]. This range covered the area of most interest for the indoor sensors, i.e., 15–50% RH and the outdoor sensors, i.e., above 40% RH. Distilled water was applied to the inorganic salts to minimize the influence of any impurities. The sensor readings during the calibration were obtained at 2–5 °C and 22 °C, for the outdoor and indoor units, respectively.

Saturated water salt solutions were placed at four 8 cm diameter plastic plates. Two of these plates were placed on the plastic box floor level and two plates were placed at about 10 cm elevation. This was done in order to quickly obtain equilibrium RH conditions after any handling of salts or weather station units in the plastic box. Air circulation within the box was achieved using a USB fan, which also speeded up the process of establishing a constant RH level. When changing to NaCl solutions, water droplets were added to an aluminum plate (for good heat transfer to the droplets [19]) placed just downstream the USB fan to quickly achieve a humid atmosphere.

The previously mentioned battery powered precision psychrometer was also placed inside the plastic container for instant recordings of temperature and relative humidity. As with the preliminary testing, a plastic rod was arranged for operating the psychrometer. The psychrometer readings were observed through the transparent plastic box lid. The box was loosely covered by sheets of aluminum foil to reflect light radiation to prevent any plastic box “greenhouse effects”. The system was then left to achieve equilibrium conditions governed by the applied saturated salt solution. The psychrometer readings were used to confirm that a constant RH level was achieved inside the plastic box. Another two hours were allowed for ensuring constant weather station readings. The temperature and RH for each weather station unit were then obtained by the web interface together with the psychrometer readings. The procedure was repeated for all the relative humidity sensors (four at a time) and for all the saturated inorganic salt solutions. Separate testing was done to establish the sensor response times when exposed to a sudden change in relative humidity.

## 4. Results

### 4.1. Calibration Coefficients for the Weather Station Relative Humidity Recordings

The sensors generally gave very erroneous results compared to the equilibrium conditions ensured by the water saturated salt solutions. Several of the detectors were up to, and even more than, 10% RH wrong either at low (11.3% RH) or high relative humidity (75.5% RH). The sensor outputs were, however, very linear with respect to the real relative humidity. This indicated that a linear equation could be used for converting the recorded result to correct relative humidity values. A linear correction curve, similar to the one shown in Equation (4), was therefore fit to the RH data recorded for the indoor units (at 22 °C) and outdoor units (at 2–5 °C). The results are presented in Table 2. The regression coefficients were better than 0.995 for all the units indicating a good straight line fit. However, when studying the slopes and intercepts presented in Table 2, it is quite clear that calibration was indeed necessary for all the sensors.

It should be noted that when extrapolating the linear curves based on the correction coefficients presented in Table 2, for some of the sensors a recorded 100% RH would turn out to yield corrected values slightly above 100% RH. This was solved by forcing any corrected result above 100% RH to 100% RH.

A number of tests were also done in the range 40% to 60% RH, where the precision psychrometer, corrected by its calibration curve, was used as the reference. No deviation from linearity was detected for any of the weather station units. To the surprise of the author, when testing an outdoor unit at indoor temperatures, the results were still within 2% RH. The calibration of the outdoor sensors at 2 °C to 5 °C was therefore taken as valid also for temperatures below 0 °C.

### 4.2. Relative Humidity Sensor Response Times

**Figure 3:**
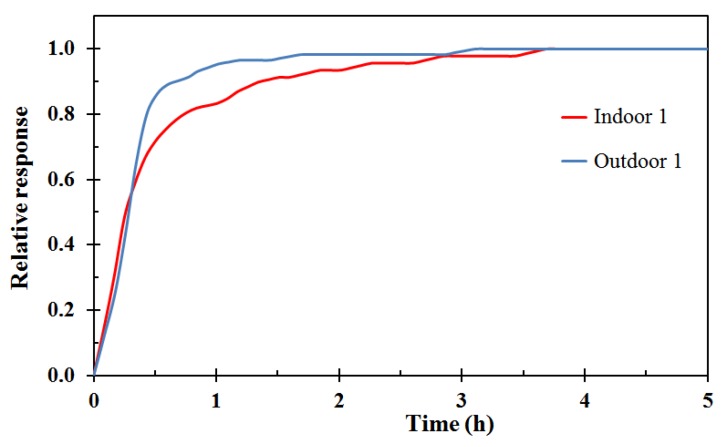
Weather station number 1 indoor (red) and outdoor (blue) sensors relative response to a near step change in relative humidity from 75% RH to 11% RH.

The sensor response time was also tested. This was done by setting the weather station number 1 indoor and outdoor units in an atmosphere stabilized by water saturated NaCl solution for 6 h. Then, the sensors were suddenly taken out and placed in the measurement container where the atmosphere was in equilibrium with water saturated LiCl solution. It should, however be mentioned that this does not represent a clean cut step function from humid (75% RH) atmosphere to dry atmosphere (11% RH) since during the sensor handling, the air in the plastic box chamber was partly diluted by indoor air of about 35% RH. Nevertheless, compared by the recording time of several hours, given the experimental setup this was as close to a step function as practicably possible. The results are shown in Figure 3, normalized such that a result of 1.0 corresponds to reaching the new constant value. The sensors did not show any change after about 3.5 h. The outdoor unit reached 95% change within 1 h while the indoor results reached 95% change towards the new constant value at just above 2 h, Whether this 2 h 95% response time was sufficient for the present work was pending the results for the measurements in real buildings, where the changes in indoor relative humidity over time was assumed to be much slower than 2 h.

### 4.3. Results Regarding Winter Fire Risk

**Figure 4:**
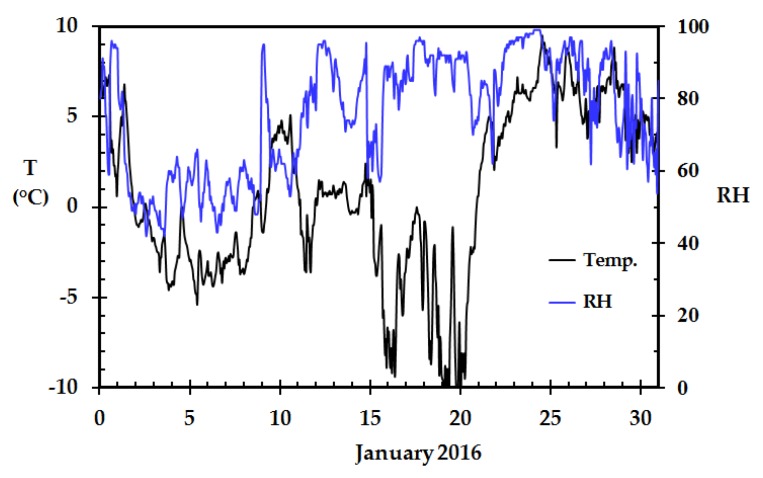
Recorded temperature and relative humidity at the local meteorological station (Haugesund airport), January 2016.

The most interesting period during the winter 2015/2016 was in January 2016. Early in January, the wind direction changed and the wind came from the mountain plains east of Haugesund, Norway. This led to adiabatically heated ambient air and lower than normal outdoor relative humidity. The recorded temperature and relative humidity from the local meteorological station at Haugesund airport for January 2016 is presented in Figure 4. 

The adiabatically heated dry subzero temperature air is easily identified in the first days of January. However, as both the temperature and the relative humidity vary, it is easier to interpret the data when studying the actual ambient air water concentration.

**Figure 5:**
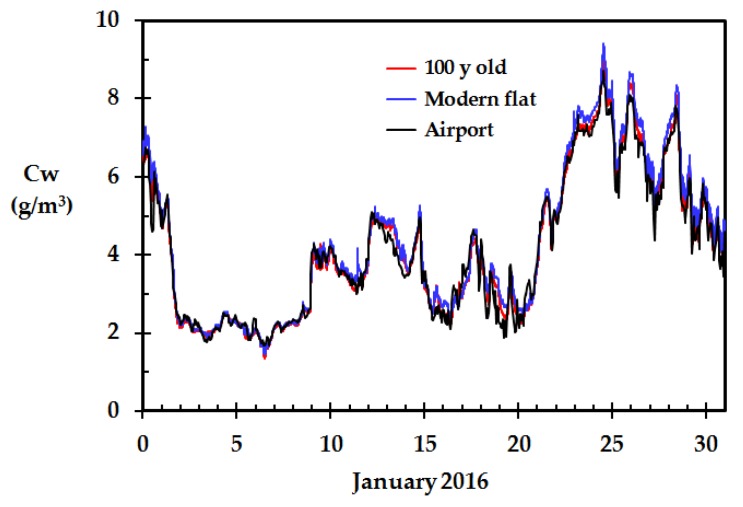
Calculated outdoor water vapor concentration for the recordings outside the 100 years old home, the modern flat and the local meteorological station (Haugesund airport), January 2016.

The water vapor concentration calculated by Equations (1) and (2), based on the Netatmo weather stations outdoor temperature and relative humidity at two homes, as well as the Haugesund airport, is shown in Figure 5. It is clearly seen from Figure 5 that the recordings at the two homes agreed very well with the recordings at the local meteorological station. This was also the case for the other six, but one, of the Netatmo weather stations. The weather station results that deviated, and a possible explanation for this deviation, are presented in Section 4.5.

**Figure 6:**
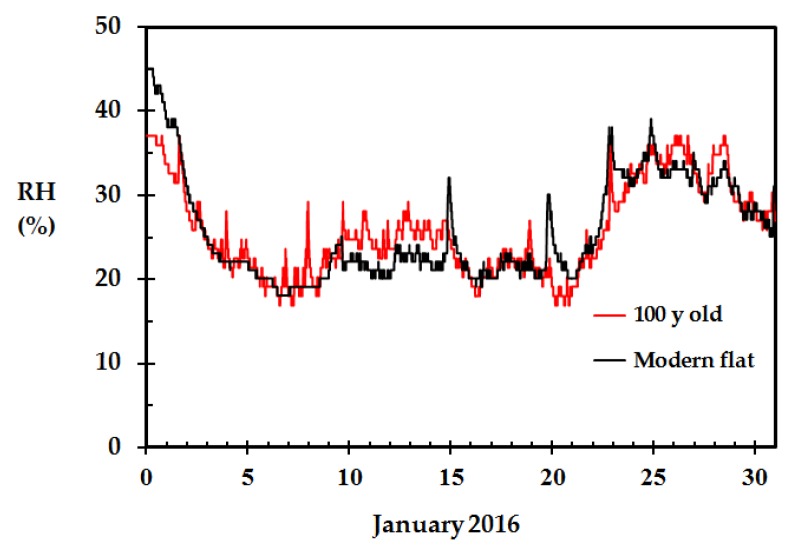
Recorded indoor RH for a 100 years old wooden home (inhabited by four persons) and a modern flat (inhabited by two persons) in Haugesund, Norway, during the cold snap in January 2016.

The indoor relative humidity recorded at the homes presented in Figure 5, is shown in Figure 6 during the January 2016 cold snap. It is seen that the indoor relative humidity was as low as 20% RH in periods before it started to increase significantly from 21 January. It is also seen that there are some indoor relative humidity peaks in both homes. Since these peaks are not synchronized in time, they are probably a result of indoor human activity.

Especially during winter time, with large outdoor to indoor temperature differences, there will be, and should be, sufficient air changes in a building to prevent rot formation in colder parts of the thermal insulation, etc. Due to the chimney effect, the indoor air will gradually be exchanged with ambient air. The ambient air entrained into the home is then heated to the indoor temperature. Wind pressure also increases the air change rate. Indoors, there will usually be some moisture gain in inhabited buildings as a result of persons and pets breathing or perspiring, dishwashing, pot plants and wood sorption processes. The wood sorption processes may go both ways, i.e., represent a positive or negative humidity gain. This also holds for other hygroscopic materials, such as furniture upholstery, pot plant soil, etc. However, assuming that there is no moisture gain, and that the ambient air is heated to the indoor conditions, a theoretical indoor relative humidity may be calculated based on the ambient conditions. The indoor weather station unit supplies the indoor temperature needed for the calculations while the ambient temperature and RH may be taken from the outdoor unit or from the local meteorological station. The best way is, however, to record the indoor RH directly, as seen in Figure 6.

It has recently been demonstrated in ¼ scale wooden compartments that the time for reaching flashover, i.e., the sudden transition from a gradually increasing fire to a fully engulfing compartment fire, was very dependent on the wood fuel moisture content (FMC). At equilibrium conditions at 20% RH, the wood equilibrium moisture content (EMC) is about 4.5% [5]. At 50% RH the EMC is about 9.3%. The ¼ scale fire testing revealed that the time to flashover when the FMC was 4.5% was just short of half the time needed to reach flashover at 9.3% FMC [19]. This dramatically reduces the margins for escape and rescue, and may in some situations be extremely critical and lead to major loss of lives [20]. Recordings like the one presented in Figure 6 may help identifying these critical situations so that proper mitigating measures may be taken for risk management.

### 4.4. Weather Forecast Data

**Figure 7:**
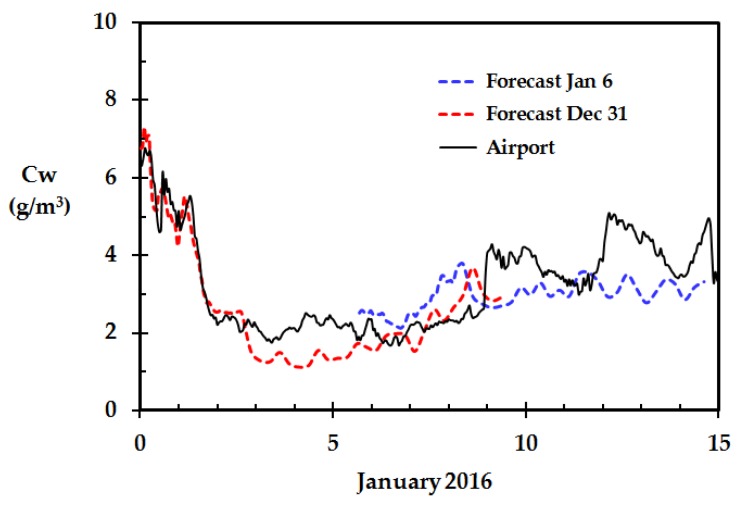
Calculated outdoor water concentration based on the forecasts at 31 December and 6 January and data subsequently retrieved from the meteorological station at Haugesund airport.

31 December 2015, at 23:00, a weather forecast API from the Norwegian Meteorological Institute was run to retrieve the forecasts for the following 10 days period. For the first 48 h, the data are detailed to each hour. Then, the data is gradually presented with less frequency. Five days later, the API was run again supplying data for the following 10 days period. The calculated ambient air water concentration based on the forecasted data is presented in Figure 7 together with the water concentration based on the subsequent recordings from the local meteorological station, i.e., the Haugesund Airport. It is evident from Figure 7 that the forecasts are quite good, at least for the first 48 h. This is very interesting when it comes to potential future predictions of the dry wood fire risk based merely on weather forecasts, as recently suggested [9]. When becoming alarmed by the weather forecast, the focus can be shifted to following up closely on selected homes with calibrated in-house weather stations, as e.g., presented in Figure 6, to confirm an increasing wooden home fire risk.

### 4.5. Other Results of Interest

**Figure 8:**
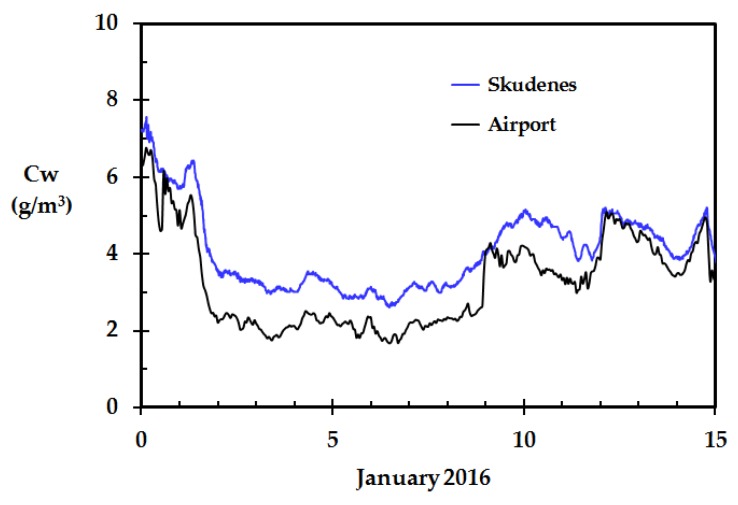
Calculated outdoor water concentration based on data from the meteorological station at Haugesund airport and data from the weather station in Skudeneshavn.

The only outdoor weather station sensor results that differed significantly from those obtained by the other sensors were from the home in Skudeneshavn, see Figure 8. This was especially the case during the first 12 days of January 2016. From 1 January to 12 January, the wind direction was about 80–100°, i.e., easterly wind from the central south Norwegian mountain plains. 12 January, the wind direction changed to about 180°, i.e., southerly wind from the open sea.

**Figure 9:**
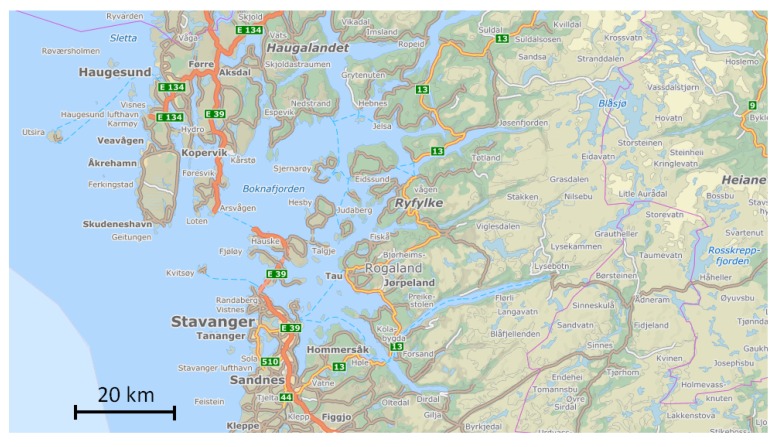
Map of the fjord Boknafjorden between Haugesund and Stavanger, Norway. North up on the map. (Accessible for free from www.norgeibilder.no).

While the other homes studied were close to the town of Haugesund, the one standing out was in Skudeneshavn, i.e., on the southern tip of the Karmøy island. As can be seen in middle left part of Figure 9, this location is more or less surrounded by sea water. 

The major fjord Boknafjorden, between Haugesund and Stavanger, has open water the whole year while the inner parts of the attached smaller fjords are covered by ice in January. In wind from east, the air passing over the open sea has a long fetch and therefore a long contact time for picking up humidity from the open water surface before arriving at Skudeneshavn. This is especially the case during winter time when the sea has a temperature well above the ambient air temperature, and when the ambient air from the east is dry due to adiabatically heating. The reason for the unit in Skudeneshavn showing the highest RH values is therefore most likely due to the dry wind from the east picking up humidity on this 25+ km long fetch during the first 12 days of January 2016.

The smaller fjord arms and most upwind lakes east of Haugesund were covered by ice during January 2016. The other weather stations close to the town of Haugesund, as seen in the upper left part of Figure 9, were therefore in the period 1 January to 12 January exposed to air that had not travelled over open and comparably warm water surfaces. When the wind direction changed on 12 January, and thereafter came from the south, all the weather stations including the one at Haugesund airport were exposed to the humid air from the sea.

## 5. Discussion

The purpose of this study was to test whether consumer grade weather stations for recording temperature and relative humidity could be used for indicating structural wooden home fire risk during winter time. The present work demonstrated that calibration was needed for the relative humidity sensor results to be within ±3% RH. The correction curves were simple linear equations. Without calibration, the readings were quite erroneous, i.e., the errors in relative humidity were up to more than 10% RH.

The calibration was done in a transparent plastic box where the air was conditioned by LiCl, MgCl_2_ and NaCl pa salt saturated water solutions. Internal air circulation was ensured by an USB powered fan. This represented a low cost setup for the relative humidity calibration. An available high precision temperature unit was used to calibrate the weather station by the built-in user based temperature calibration app. The recorded temperatures were well within 0.3 °C when tested after this user calibration. Using the high precision temperature unit may, however, be seen as an overkill since a normal type K thermocouples would probably have been sufficiently accurate for the temperatures of interest in present work.

The precision psychrometer was used to check that the inorganic salts did indeed produce the correct relative humidity. This was also in principle not necessary, as the saturated salt solutions would ensure equilibrium conditions at a known relative humidity level [18]. The psychrometer did, however, represent an independent way for checking that the correct saturated salt solution was applied and gave a good indication about the time needed to achieve the actual equilibrium conditions.

The selected weather stations had a built-in web application which allowed for remote retrieval of the data recorded at 5 min intervals. The recorded data was downloaded in the form of spread sheets. It was therefore easy to apply the proper relative humidity correction equation for each indoor and outdoor detector unit.

The tested weather stations had a resolution of 1% RH. This made it difficult to detect any weather station hysteresis regarding the relative humidity recordings. The weather station response was slow, i.e., a time period of about 2 h was therefore allowed to ensure correct results. The sensors can therefore not be used in situations where a fast response time is required. When the ambient water vapor concentration decreased considerably in January 2016, it was seen that a typical home needed a few days to equilibrate to the new and drier condition, as seen in Figure 6.

In principle, the measurement frequency of 12 h^−1^ allows for detecting quite rapid changes in the indoor relative humidity, e.g., as a result of airing. However, given the slow response of the units, i.e., close to two hours, any such potential sudden changes in indoor relative humidity could not be discovered. Peaks due to cooking activities were, however, discovered. Due to the slow response, the peak values were probably not reliable as the sensor recordings were probably lagging behind such rather rapid changes in indoor relative humidity.

The weather station response time must be compared to the building response time, i.e., a few days. The conservative two hours weather station response time was an order of magnitude faster than the building indoor response time. This was taken as a proof of the sensor response time being sufficiently fast for the purpose of the present work, i.e., demonstrating that consumer grade weather stations can be used to monitor the dry indoor climate fire risk levels of wooden buildings.

It was clearly seen that the indoor sensor had a longer response time compared to the outdoor sensor when tested under the same conditions, i.e., 2 h versus 1 h to 95% of a step change in relative humidity. This may simply be due to the larger internal air volume of the indoor sensors. For future measurement campaigns it is recommended to try to optimize the response time by e.g., allowing for better air convection and thereby faster response.

During the calibration, the outdoor relative humidity sensors were, as always, powered by internal batteries. The indoor relative humidity sensors were, however, supplied USB power from transformers outside the box, i.e., the current wires had to be led through the wall. This was also the case for the USB fan power cable. Instead of relying on external power, using USB power banks for supplying these units would take away the clutter of wires through the wall. Utilizing USB power banks as the power supply source are therefore recommended for future calibration and characterization studies.

The accuracy of the recordings, after the calibration curves were established and applied to the retrieved data, was found to be within ±3% RH. This was also the case for three weather stations that were tested after four months of operation during December 2015 through March 2016. This accuracy was still well within the goal for the present study. Advanced relative humidity sensors may display results far better than this, and with response times of 35 s [10] or even as low as 2 s [11]. Such improved relative humidity sensors may become available on the consumer market. But for now, as no dry wood fire risk warning system exists, the weather stations tested in the present work was found fit for purpose. They did work quite well as an indicator of dry indoor climate resulting in a gradually increasing wooden home structural fire risk. The long time stability through winter and summer season was not tested in the present work since, based on the simplicity of the inorganic salt calibration, the detectors will regardless be recalibrated for future measurement campaigns.

An interesting research possibility regarding web based weather stations may be related to the geography teaching for primary and secondary schools. The influence of the sea and the mountains on the local relative humidity, as clearly observed in the present work, may be demonstrated in the class room. The changes in relative humidity when the temperature is changing may also be worthwhile presenting to school children. The calibration procedure for teaching purposes may be done without using pro analysis quality salts. Commercial grade NaCl is available in any grocery store, though maybe producing equilibrium conditions 1% RH off due to a minor content of other salts. Consumer grade MgCl_2_ may be available at hardware stores as an ice melting powder, at least in cold climate areas. MgCl_2_, which in contrast to LiCl, is not poisonous, may therefore represent a safe second calibration point.

Another research possibility may be to use private weather stations for suppling data for increased wildfire risk awareness, especially during summer time [21], but also during winter time in cold climate areas [6]. Recent wildland urban interface (WUI) fires in Europe have also claimed many lives, such as the one in Portugal 16–24 June 2017, which claimed 66 lives [21]. The Athens area WUI fires during 20–22 July 2018 resulted in more than 90 fatalities [22]. If the public becomes more aware of the overhanging wildfire and wildland urban interface fire risk by reading properly calibrated ambient relative humidity data, they may be better prepared for preventing igniting start fires as well as for an early evacuation if a wildfire starts in their region. Increasing the risk awareness using e.g., phone app warnings based on private weather stations may be a worthwhile future research project.

The main conclusion of the present study is that consumer grade weather stations, at least the type tested in the present study, have been shown to quite accurately measure the indoor and outdoor relative humidity when properly calibrated. This indicates that such weather stations could become central in a future attempt to develop a cold climate structural fire risk warning system.

## Figures and Tables

**Table 1:** Producer calibration data for the Extec RH390 precision psychrometer (in a Binder KBF-115 climate chamber, serial No. 09-06299, Binder GmbH, Ulm, Germany, traceable to Scalibra AS, Skjetten, Norway, proof No. 3491-14).

RH_real_	RH_reading_	Equation (4) Deviation
24.0%	23.7%	−0.05%
49.5%	48.5%	0.10%
75.0%	73.0%	−0.05%

**Table 2:** The linear correction curve for the eight weather stations studied.

Sensor	Slope	Intercept
Indoor 1	1.030	−7.778
Outdoor 1	1.092	−6.334
Indoor 2	1.074	−10.811
Outdoor 2	1.090	−6.734
Indoor 3	1.122	−16.707
Outdoor 3	1.108	−7.464
Indoor 4	1.045	−11.716
Outdoor 4	1.118	−8.694
Indoor 5	0.991	−7.713
utdoor 5	1.107	−7.973
Indoor 6	0.935	−4.655
Outdoor 6	1.1051	−4.826
Indoor 7	0.999	−7.872
Outdoor 7	1.103	−6.420
Indoor 8	0.989	−8.778
Outdoor 8	1.138	−9.091
